# High sensitization efficiency and energy transfer routes for population inversion at low pump intensity in Er organic complexes for IR amplification

**DOI:** 10.1038/s41598-018-21700-7

**Published:** 2018-02-19

**Authors:** J. X. Hu, S. Karamshuk, J. Gorbaciova, H. Q. Ye, H. Lu, Y. P. Zhang, Y. X. Zheng, X. Liang, I. Hernández, P. B. Wyatt, W. P. Gillin

**Affiliations:** 1 College of Physical Science and Technology, Sichuan University, Chengdu, 610064 China; 2 Materials Research Institute and School of Physics and Astronomy, Queen Mary University of London, Mile End Road, London, E1 4NS UK; 3 Materials Research Institute and School of Biological and Chemical Sciences, Queen Mary University of London, Mile End Road, London, E1 4NS UK; 4 Division of Physics and Applied Physics, School of Physical and Mathematical Sciences, Nanyang Technological University, 21 Nanyang Link, Singapore, 637371 Singapore; 5 State Key Laboratory of ASIC and System, SIST, Fudan University, Shanghai, 200433 China; 6 State Key Laboratory of Coordination Chemistry, Nanjing National Laboratory of Microstructures, School of Chemistry and Chemical Engineering, Nanjing University, Nanjing, 210093 China; 7 Dpto. CITIMAC, Facultad de Ciencias, Universidad de Cantabria, Avda. Los Castros, s/n, 39005 Santander, Spain

## Abstract

Organic erbium complexes have long been of interest due to their potential for using the strong absorption into the organic to sensitise the erbium emission. Despite this interest there is remarkably little quantitative information on how effective the approach is and the discussion of the energy transfer mechanism is generally vague. Here we accurately quantify the sensitisation as a function of excitation pump density and model it using a rate equation approach. As a result, we can calculate the degree of population inversion for the erbium ions as a function of the pump intensity. We demonstrate that even when we increase the erbium concentration in the films from ~10 to ~80% we find a relatively small decrease in the sensitisation which we attribute to the large (>20 Å) Förster radius for the sensitisation process. We show that we can obtain population inversion in our films at very low pump powers ~600 mW/cm^2^. The calculated Förster radius for the organic erbium complexes suggests design rules for energy transfer between antennas and erbium ions in molecular systems and hybrid organic-inorganic nanoparticles.

## Introduction

Erbium has long been of interest as a near infrared (NIR) emitter as its emission wavelength (1.5 µm) matches perfectly to the low loss transmission wavelengths for silica optical fibres^1–4^. It is extensively used in the erbium doped optical amplifier which is the technology that underpins the global fibre optic networks that are vital for long-distance broadband data transmission^5,6^. In these systems the erbium ions are doped into silica glass but due to the poor solubility and a low absorption cross section for the erbium ions the amplifiers need long gain regions and high power pump lasers (typically ~100 kW/cm^2^). Sensitisation of erbium ions to improve their effective absorption cross section has been used for many years and the most successful sensitizer has been to co-dope erbium ions with ytterbium^7–10^. This has an absorption cross section at ~980 nm that is about an order of magnitude greater than that of erbium and once excited the ytterbium very efficiently transfers its energy to nearby erbium ions. However, even with ytterbium as a sensitizer it is still necessary to use lasers to achieve the high excitation densities required for population inversion.

It has long been suggested that lanthanide ions could be sensitised by incorporating them into an organic host and using the “antenna effect” where the pump light is absorbed by the organic moiety and the energy is efficiently transferred to the lanthanide^11–13^. As the absorption by organic ligands is typically 10^6^ times greater than that of erbium this approach has the potential to dramatically improve the effective absorption cross-section for the ions. In addition, it also allows for the possibility of increasing the maximum erbium concentration by effectively removing the solid solubility limits and providing precise control on the inter-ion distance which can be used to remove ion-ion interactions. Using organics it is possible to achieve erbium concentrations of up to 4.5 × 10^20^ cm^−3^ (the highest concentration used in this study) which is up to an order of magnitude greater than can generally be achieved in glasses^14^. When this is coupled with the increase in the effective absorption cross section this results in an approach that can lead to high gain in very compact devices with low intensity pump sources such as low-cost LEDs. Ultimately these materials can be integrated directly onto silicon photonic circuits and can provide distributed gain to counter the intrinsic losses in those structures but more excitingly they offer the possibility of producing a cost-effective silicon laser. Given that the organic molecules used are semiconductors then this also leads to the possibility of producing integrated laser devices that can be electrically pumped.

Despite several decades of research there is still little information as to how efficient the sensitisation of erbium by an organic host can be^15–17^. Much of the work on organic erbium materials has been hampered by the exceptionally low quantum efficiencies for erbium emission, which is caused by the vibrational quenching through high-energy oscillators of CH, OH and NH bonds in the organics^18–20^. Regardless of the low quantum efficiencies that have commonly been achieved in erbium organic materials it is still the case that most of the published work merely demonstrates that some energy transfer must be occurring between the organic and the erbium with few attempts to fully quantify it^21^. In recent works we have used excitation spectroscopy to give some quantification of the integrated sensitisation and demonstrated that it can range from the very low (factor of ~5)^22^ to the very high (~10000)^23^. However, the use of excitation spectroscopy only tends to provide data over a limited range of excitation intensities and can be prone to error at high sensitisation due to the difficulty in observing the direct absorption into the ion. In this work, we perform quantitative measurements of the erbium emission intensity as a function of pump power using two different wavelengths. One pump laser is at 655 nm so that it is solely absorbed by the ^4^I_15/2_ to ^4^F_9/2_ transition in the erbium ions whilst the second is at 407 nm which is solely absorbed by the organic chromophore. For the organic erbium complex we used erbium(III) tetrakis(pentafluorophenyl)imidodiphosphinate^24^, (Er(F-TPIP)_3_), which we have demonstrated can have a quantum efficiency of ~7% when deposited by vacuum sublimation in a vacuum of ~10^−7^ mbar. For the chromophore we utilised the zinc(II) salt of 2-(3,4,5,6-tetrafluoro-2-hydroxyphenyl)-4,5,6,7-tetrafluorobenzothiazole, Zn(F-BTZ)_2_, which we have already shown can have efficient energy transfer to the erbium ions^23^. We have quantified the sensitisation in a series of films as a function of molar erbium concentrations and developed a simple rate equation model that allows us to not only quantify the sensitisation but also calculate the population of erbium ions in the excited state as a function of excitation pump power. Using this model, we have demonstrated that we can achieve population inversion for the erbium ions at excitation levels as low as 500 mW/cm^2^ and that with a modest increase in the quantum efficiency of the erbium to 50% this power requirement can be reduced to only 40 mW/cm^2^. We have also calculated the Förster radius for the sensitization process and discuss their implications for energy transfer at a nanoscale level.

## Results and Discussion

**Figure 1:**
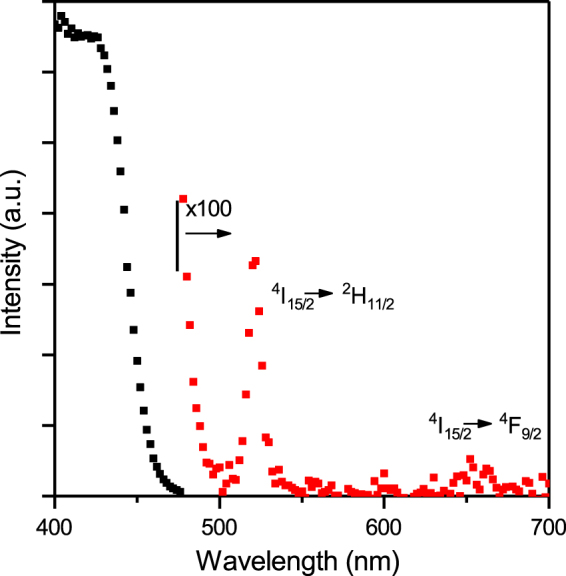
The 1535 nm excitation spectrum for the Zn(F-BTZ)_2_ film doped with 9% Er(F-TPIP)_3_. Data at wavelengths longer than 475 nm have been scaled by a factor of 100. A weak direct absorption into the ^4^F_9/2_ level can just be observed.

In Fig. 1 we show the excitation spectrum for the co-doped film recorded for the ^4^I_13/2_ to ^4^I_15/2_ transition at 1535 nm. This was recorded using a monochromated Xe lamp as an excitation source and it can be clearly seen that the excitation through the ligand is dramatically stronger than the direct absorption into the ^4^I_15/2_ to ^2^H_11/2_ (~520 nm) and ^4^I_15/2_ to ^4^F_9/2_ (~655 nm) Er^3+^ levels. There is still a weak tail from the ligand excitation at 520 nm and hence we have used the ^4^I_15/2_ to ^4^F_9/2_ at 655 nm for the detailed sensitisation measurements. Due to the weak direct absorption into erbium in the visible it is often difficult to obtain high quality excitation spectra for the direct absorption unless there is a high concentration of erbium ions in the sample. This can make the quantification of the sensitisation using this method prone to error.

**Figure 2:**
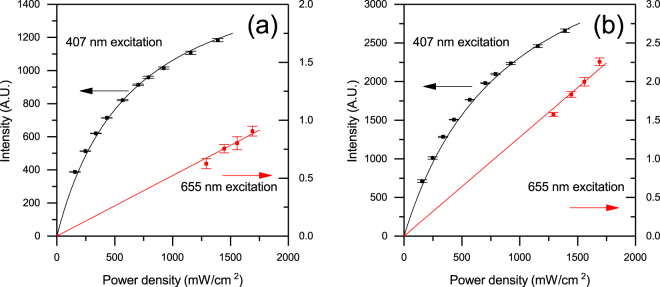
The intensity of the Er ^4^I_13/2_ to ^4^I_15/2_ transition at 1530 nm as a function of the pump laser power density for Zn(F-BTZ)_2_ films doped with (**a**) 9% Er(F-TPIP)_3_ and (**b**) 23% Er(F-TPIP)_3_. The black data are for the 407 nm excitation and the red data (right hand axis) are for the 655 nm excitation. The solid lines are the results of the rate equation modelling.

**Figure 3:**
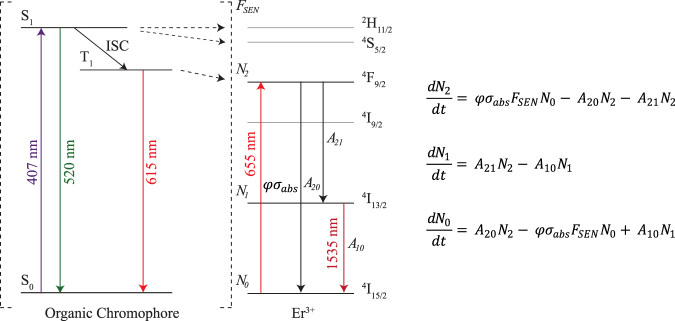
Jablonski diagram for the energy transfer between the chromophore and the erbium ion. The rate equations are based on the simplified energy level diagram using only the ground, first and third excited states. Sensitisation is included simply through a sensitisation factor, *F*_*SEN*_, which is a multiplier for the absorption from the ground state to the third excited level.

In Fig. 2a we plot the erbium emission intensity as a function of the incident power density for a 9% doped Er(F-TPIP)_3_-Zn(F-BTZ)_2_ under both 655 nm and 407 nm excitation. Under 655 nm excitation we are only able to measure the Er emission at large pump power densities due to the weak absorption of the erbium in the thin films. It can be seen that the emission intensity is linear with the excitation pump density. For 407 nm excitation we can detect the erbium emission intensity at much lower power densities and it can be seen that the Er emission tends to saturate with increasing pump power. The solid lines are a fit to the data using a simple rate equation approach where we take the erbium ion as being a three level system consisting of the ground state (^4^I_15/2_), the emissive first excited state (^4^I_13/2_) and the directly excited state (^4^F_9/2_). In Fig. 3 we provide a Jablonski diagram for the system along with the simplified energy level diagram and the corresponding rate equations used to model the results. For the rate equations only three levels are taken into account: the ground state (^4^I_15/2_) of the Er^3+^, (with a population of *N*_0_), the first excited state (^4^I_13/2_) of the Er^3+^, (with a population of *N*_1_), and the third excited state (^4^F_9/2_) of the Er^3+^, (with a population of *N*_2_). The transition rate, A_20_, (^4^F_9/2_ to ^4^I_15/2_) was taken as 1000 s^−1^ and was obtained from a Judd-Ofelt calculation for Er(F-TPIP)_3_^25^. The transition rate A_21_ (^4^F_9/2_ to ^4^I_13/2_) (τ_21_ = 0.5 µs) was measured directly from the rise time for the ^4^I_13/2_ to ^4^I_15/2_ transition when the ^4^F_9/2_ level was excited directly using an ~5 ns pulse at 655 nm from an OPO. The initial population of ions in the ground state (*N*_0_) is known from the Er concentration in the films. The decay lifetime for the ^4^I_13/2_ to ^4^I_15/2_ transition was measured directly as a function of 407 nm excitation intensity for each sample. In every case the decay was found to be bi-exponential with a long lifetime component of ~700 ± 50 µs and a short component of ~400 ± 50 µs. When we directly excited the erbium in these films with an ~5 ns pulse at 520 nm we always observed mono-exponential decay with the 700 µs decay component. This implies that the 400 µs component is a function of the very high excitation conditions that occur with continuous illumination and high sensitisation. The average lifetime of this transition generally shows a small (<10%) linear decrease with increasing power density caused by a shift in the relative populations of the two lifetime states. There was no systematic variation in lifetimes as a function of the erbium concentration. The value of *A*_10_ we used in our model was therefore determined from a linear fit to this average lifetime and hence was a function of the excitation intensity. The pump rate, *R*_*P*_, is given by the absorption cross-section for the ^4^I_15/2_ to ^4^F_9/2_ (σ_abs_ = 1.3 × 10^−21^ cm^2^) multiplied by the photon flux density (φ)^25^. The fit to the 655 nm data only has a single scaling factor in it to convert the emission rate from the model (*N*_1_*A*_10_) into the arbitrary units of the intensity measured by the photomultiplier. As the experimental setup is identical under 655 nm and 407 nm excitation this scaling factor remains constant.

From Fig. 2a it can be seen that the fit to the 655 nm data shows a straight line passing through the origin as one would expect, considering the low absorption cross section for erbium at this wavelength which results in a low concentration of erbium ions in the excited state. The error bars show the statistical error as we repeat each measurement 10 times. For the 407 nm excitation the power density plotted is the actual power density absorbed by the chromophore and we ignored all the detail of the energy transfer processes both within the chromophore and between the chromophore and the erbium ion and simply approximated this by a sensitisation factor, *F*_*SEN*_, which is a multiplier to the absorption cross section of the ^4^I_15/2_ to ^4^F_9/2_ transition. This does not mean that the sensitisation is actually going through this level, only that it can be modelled as an effective increase in the absorption coefficient for the erbium. This is a very simple approach which does not take into account any competing process that may be occurring within the sensitizer (such as triplet-triplet annihilation at high pump powers). The solid line through the 407 nm data in Fig. 2 is the result of this model using a sensitisation factor of 5200 and shows that this simple model provides an excellent fit to the experimental data. The output of the rate equation approach is the population of each level as a function of excitation power and we multiply the population of the first excited state (*N*_1_) by the transition rate, *A*_10_, to obtain the luminescence intensity from the erbium. As the value of *A*_10_ is almost constant over the range of excitation powers used (and the small variation is taken into account in our model) the sublinear shape of the 407 nm excited emission is a direct measure of the population of ions that are in the excited state (*N*_1_). We are therefore able to directly calculate the ratio of erbium ions in the excited state compared to those in the ground state (*N*_0_) and can determine that in the 9% sample we have achieved population inversion (50% of the ions in the excited state) at a pump density of ~600 mW/cm^2^. For the 23% doped Er(F-TPIP)_3_-Zn(F-BTZ)_2_ film (Fig. 2b) we obtained a similar excellent fit to the data with a calculated sensitisation factor of 3900. This reduction in the sensitisation factor is probably an effect of the lower concentration of chromophore molecules available to excite the erbium ions.

**Figure 4:**
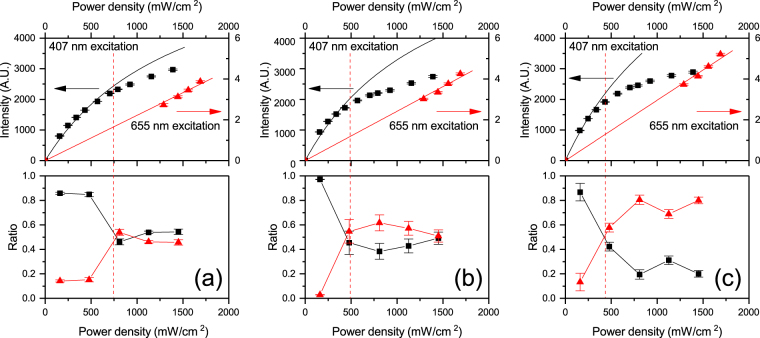
Top panel is the intensity of the Er ^4^I_13/2_ to ^4^I_15/2_ transition at 1530 nm as a function of the pump laser power density for the (**a**) 44%, (**b**) 64% and (**c**) 81% Er(F-TPIP)_3_ films. The black data are for the 407 nm excitation and the red data (right hand axis) are for the 655 nm excitation. The solid lines are the results of the rate equation modelling. In the bottom panel is the ratio of the long and short component of the Er ^4^I_13/2_ to ^4^I_15/2_ transition lifetime in the same samples. The black data are the proportion of the long component and the red data the proportion of the short component.

Figure 4 shows the equivalent data for the 44%, 64% and 81% doped Er(F-TPIP)_3_-Zn(F-BTZ)_2_ films. The sensitisation factors used to achieve the fits shown are 2500, 2400 and 2200 for the 44%, 64% and 81% films respectively. In each of these films we can see that our model works at low excitation densities but the experimental data fall below the model as the pump power density increases. Given that the concentration of the Zn(F-BTZ)_2_ chromophore is decreasing it is unlikely that this is due to some decrease in the number of excited states (triplets) present in these films with increasing excitation intensity as the probabilities of likely quenching processes (e.g. triplet-triplet annihilation) are reduced at lower chromophore concentrations. Similarly, we have kept the total number of Zn(F-BTZ)_2_ molecules constant in each film so it cannot be due to a lower absorption by the chromophore. In addition as each chromophore is effectively in the presence of a greater number of erbium ions it is unlikely to be due to there not being enough erbium ions present to be excited in these films. As can be seen in Fig. 4, the deviation from the model gets worse as the erbium concentration increases; this implies that there must be additional de-excitation routes for the erbium ions at higher concentrations. It is well known that as the Er^3+^- Er^3+^ separation decreases the probability for inter-ion quenching increases^4^. We noted earlier that the erbium lifetime shows a bi-exponential decay with a long and short lifetime component and that there is a shift from the population with the longer lifetime to the shorter one as the pump power increases. In Fig. 4 we plot the relative contributions of the two lifetime components as a function of the excitation power. For the 9% film the long lifetime component accounts for nearly 100% of the emission at low excitation pump powers and decreases to ~80% at the maximum pump power. For the 23% film the long component accounts for ~80% at low excitation powers and this reduces to ~60% at the maximum pump power. However, for the films with 44% Er concentration and higher a different behaviour is observed. For the 44% film at low excitation powers the long component accounts for >80% of the emission but at an excitation power density of ~1000 mW/cm^2^ this reduces to ~50% and remains at that level as the pump power is increased. This change in the erbium decay time corresponds to the point where the experimental data start to deviate from the model (~790 mW/cm^2^). For the 64% film the effect is even more dramatic with the long component decreasing from ~100% at low power to 50% at ~600 mW/cm^2^ and for the 81% film the long component shows a similar effect but keeps reducing to ~20% at a pump power of ~1000 mW/cm^2^. Again these changes in the erbium decay lifetimes correspond to the power density where the experimental data start to deviate from the model. This correlation between the deviation of the 407 nm excited data from the model and the observed reduction in the lifetime of the erbium ions in the film suggests that with the higher erbium concentration films we are starting to observe ion-ion quenching with increasing pump power. Although this effect is taken into account in the measured decay time as a function of excitation intensity the model does not account for the fact that ion-ion interactions can effectively quench more than one ion in a single process (One example of this could be cooperative up-conversion between two excited erbium ions followed by energy transfer back to the chromophore) and hence the number of excited erbium ions in the sample falls below the model.

From the rate equation model, we can determine the percentage of ions in the first excited state relative to the ground state as a function of excitation intensity. Using this model, we can determine that the deviation of the experimental data from the model occurs when the population of excited ions in the layer reaches ~30% (35%, 24% and 21% for the 44%, 64% and 81% Er(F-TPIP)_3_ films respectively). The fact that these relatively high populations in the excited state are needed to see a reduction in the Er emission lifetime also supports the proposal that this is probably due to ion-ion interactions. As the model directly gives us the populations in the different levels we can use it to determine the pump power density where we see the onset of population inversion (<50% of ions in the ground state). For our 9% and 23% Er(F-TPIP)_3_ films we find that this occurs at pump power densities of 620 mW/cm^2^ and 850 mW/cm^2^ respectively. These values are remarkably low and within the range that can easily be obtained using a conventional LED as a pump source. If we were able to increase the quantum efficiency of the erbium ions to 50% (For example by reducing quenching caused by residual impurities) then we would be able to reduce the pump power needed for population inversion to ~50 mW/cm^2^ and ~70 mW/cm^2^ respectively. This would be within the range that can easily be achieved by using an OLED as the pump source and would open the possibility of fabricating a purely organic, electrically driven device.

**Figure 5:**
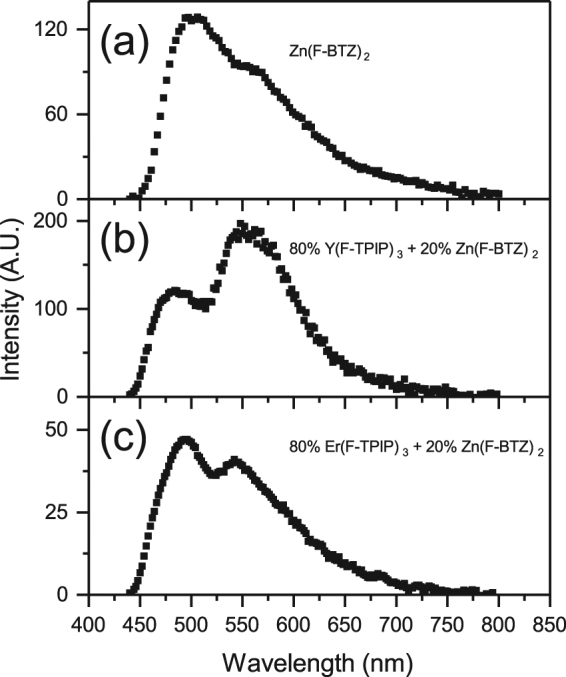
The 80 K photoluminescence from (**a**) a 200 nm film of Zn(F-BTZ)_2_, (**b**) 200 nm of Zn(F-BTZ)_2_ co-doped into a 80% Y(F-TPIP)_3_ and (**c**) 200 nm of Zn(F-BTZ)_2_ co-doped into a 80% Er(F-TPIP)_3_.

**Figure 6:**
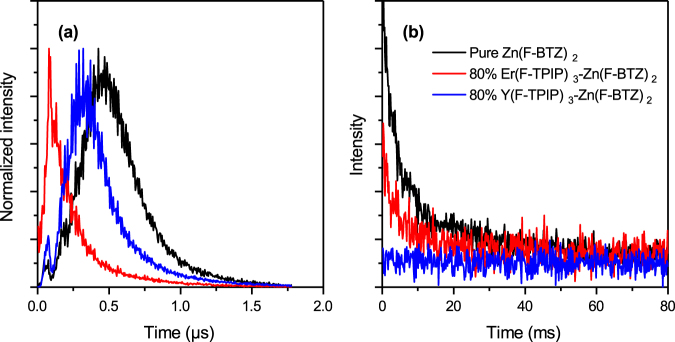
Time evolution of the singlet emission (**a**) and triplet emission (**b**) from delayed fluorescence measurement conducted on the pure Zn(F-BTZ)_2_ film, 80% Y(F-TPIP)_3_-Zn(F-BTZ)_2_ film and 80% Er(F-TPIP)_3_-Zn(F-BTZ)_2_ film under 80 K.

It is generally assumed that the major energy transfer mechanism between an excited state on an organic molecule and a lanthanide ion is via the triplet state of the organic and hence that photoexcited molecules must undergo intersystem crossing (ISC)^26^. The Zn(F-BTZ)_2_ chromophore used in these experiments has enhanced ISC, compared to the hydrogenated version, due to the effect of the large number of fluorine atoms that have replaced the hydrogen. This can be seen in the low temperature photoluminescence of the material (Fig. 5a) which shows a distinct triplet emission. For a 200 nm film of Zn(F-BTZ)_2_ we measured an 80 K recombination lifetime for the triplet of ~20 ms. This film showed distinct delayed singlet emission following excitation with a ~5 ns pulse which peaked about 500 ns and decays after about 2 µs (see Fig. 6). This delayed fluorescence is indicative of triplet-triplet annihilation within the layer. We therefore prepared a sample where the same quantity of Zn(F-BTZ)_2_ is co-evaporated into an optically inert Y(F-TPIP)_3_ matrix so that the final layer has the molar concentrations of 80% Y(F-TPIP)_3_ and 20% Zn(F-BTZ)_2_. This has the effect of separating the Zn(F-BTZ)_2_ molecules so as to reduce triplet quenching interactions. As a result, the photoluminescence, shown in Fig. 5b is now dominated by emission from the triplet which has a lifetime of ~120 ms. In this film. the absolute photoluminescence intensity of the singlet emission is reduced by ~10% from that in the pure film whilst the intensity from the triplet emission is ~60% greater than that for the singlet. These spectra were all recorded by integrating the area under the decay curve after excitation with a 50 ms laser pulse (at a repetition rate of 0.27 Hz). Due to the long lifetime for the triplet, coupled with the relatively high ISC rate, these long laser pulses allow time for a build-up in the triplet population. Figure 5c shows the photoluminescence from a similar film where the Zn(F-BTZ)_2_ has been diluted with 80% Er(F-TPIP)_3_. Here the dilution means that the self-quenching of the Zn(F-BTZ)_2_ is reduced as in the Y(F-TPIP)_3_ doped film. However, in the Er(F-TPIP)_3_ doped film the triplet emission intensity is only 25% of that measured in the Y(F-TPIP)_3_ doped film and the singlet emission is reduced by over 50%. The triplet lifetime is only ~60 ms in this film. These results demonstrate that for Zn(F-BTZ)_2_ as a sensitizer of Er(F-TPIP)_3_ both singlets and triplets couple very efficiently into the Er^3+^ ions. The fact that the singlet lifetime in this material is only of the order of 10 ns shows how efficient this energy transfer must be for ~50% of the singlets to be quenched. Using Equation 1 we have calculated the Förster radius, *R*_0_, for the singlet to couple to the Er^3+^ ion.

1 $${R}_{0}^{6}=\frac{9000\,\mathrm{ln}\,10}{128{\pi }^{5}{N}_{A}}\frac{{\kappa }^{2}{Q}_{D}}{{n}^{4}}\int {f}_{D}(\lambda ){\varepsilon }_{A}(\lambda ){\lambda }^{4}d\lambda $$

**Figure 7:**
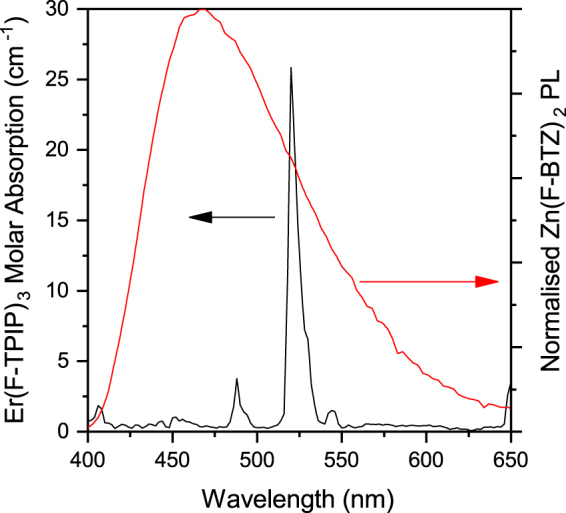
The molar absorption spectrum for Er(F-TPIP)_3_ and the room temperature photoluminescence spectrum for Zn(F-BTZ)_2_ which are used to calculate the Förster radius for energy transfer between the chromophore and the Er ions.

Where *N*_*A*_ is Avogadro’s number, *κ*^2^ is the dipole orientation factor which we have taken as 2/3, *Q*_*D*_ is the quantum yield of the donor which we have assumed to be unity, *n* is the refractive index of the medium (1.49 for Er(F-TPIP)_3_ and 1.60 for Zn(F-BTZ)_2_ both at 633 nm), *f*_*D*_*(λ)* is the normalised donor emission spectra (given in Fig. 7 for Zn(F-BTZ)_2_) and ε_A_(λ) is the acceptor molar absorption coefficient (also given in Fig. 7 for Er(F-TPIP)_3_). We do not rule out the possibility for Dexter transfer occurring in these films but the fact that our erbium ions are in one molecule (with a radius of ~7 Å) and the chromophore is a separate molecule, and not necessarily the next nearest neighbour, means that they are physically separated and hence the possibility of there being significant overlap of their wave-functions is reduced. For the singlet emission shown in Fig. 6 we obtain a Förster radius of ~21 Å whereas due to the improved spectra overlap this increases to 25 Å when the triplet emission is included. As the Förster radius is defined as the distance at which there is a 50% probability of energy transfer from the donor to the acceptor and given that the “radius” of an Er(F-TPIP)_3_ molecule is ~7 Å this demonstrates that if the chromophore is in a nearest neighbour location the coupling should be very strong whereas even in a second nearest neighbour location there should still be very good coupling. We have seen a 50% reduction in both the singlet intensity and the triplet lifetime in the 80% Er(F-TPIP)_3_ doped film. This should correspond to the chromophore molecules being >20 Å from any Er ions which is obviously not true in the 80% doped films. Therefore, one might expect the observed quenching to be much higher. However, the excitation power density used in these films is of the order 300–600 mW/cm^2^ (10 mW laser intensity and a spot diameter of ~1.5–2 mm); at room temperature this was sufficient to have ~20% of the Er^3+^ ions in the excited state and these ions are going to be the ones that are closest to the chromophore molecules. Therefore, under these large excitation intensities it would be expected that chromophores will excite Er ions efficiently and then be re-excited by the pump and that once the nearest Er ions have been excited the chromophore molecules will no longer be quenched. It is therefore the case that at this excitation density there are extra excited states within the Zn(F-BTZ)_2_ to replenish Er^3+^ that are de-excited. This would be a fundamental requirement for maintaining gain in a laser application.

An alternative approach to using organic sensitizers with IR emitting lanthanides has been to couple them to doped inorganic nanoparticles^27–30^. This type of composite based on inorganic nanoparticles also have organic groups on the surface and can be dispersed in organic solvents. Effective protection the lanthanide from the organic environment by nanoparticles has been achieved so as to produce relatively long infrared lifetimes^29^. The size of nanoparticles (typically larger or of the order of ~10 nm) is probably the main obstacle to achieve efficient sensitization, as we have shown that the Förster energy transfer from a visible chromophore into erbium operates in the range of ~2 nm which limits sensitisation into the core of the nanoparticle^29^. Nevertheless, further reduction of nanoparticle size will also lead to increased quenching of the erbium ions through interaction with OH and CH groups on the surface.

In conclusion, we have demonstrated a method for accurately determining the sensitisation of erbium ions in an organic host through a chromophore. We have shown that the sensitisation can be easily modelled as a simple scaling of the absorption cross section for the ion provided that ion-ion quenching interactions can be removed by keeping the Er(F-TPIP)_3_ concentration below ~44%. Using this approach we have been able to accurately quantify the sensitisation to a value of 5200 for a 9% film and 3700 for a 23% film and we find that the sensitisation saturates at ~2200 for erbium concentrations from 44 to 81%. This result is particularly exciting as it means that it is possible to get significant increases in excited erbium atoms in a film without further reduction in the sensitisation provided by the organic. If we were able to reduce the ion-ion quenching by using bulkier ligands that increase the spacing between erbium ions then we might be able to remove the ion-ion quenching with only a modest reduction in the erbium concentration whilst keeping a very high sensitisation factor. The model has also allowed us to demonstrate that we can obtain population inversion in these layers at very low optical power densities that are well within the range that can be achieved over large areas using conventional LED illumination. We have also demonstrated that both the singlet and triplet states of the chromophore can effectively couple into the Er^3+^ ions but due to their longer lifetimes it would be best to use chromophores with higher ISC rates in order to provide a reservoir of excited states to replace Er^3+^ ions that are de-excited to their ground state.

## Methods

Co-doped films of Zn(F-BTZ)_2_ and Er(F-TPIP)_3_ were deposited by vacuum sublimation at a base pressure of ~10^−7^ mbar onto a glass substrate. The layers were then encapsulated with a 100 nm film of aluminium to protect the material from atmospheric degradation. Samples were prepared with Er(F-TPIP)_3_ mole fractions of 9, 23, 44, 64 and 81% and each film was grown with an identical amount of the Zn(F-BTZ)_2_ chromophore (50 nm effective thickness) in order to ensure that the absorption of the 407 nm laser was constant for each film. 50 nm was chosen as this thickness (coupled with the reflective Al capping layer) ensured that the intensity of the light absorbed was relatively uniform (~20% variation) through the film. In order to ensure consistent and reproducible absorption and emission detection between samples, the two different pump lasers were made co-axial and brought in normal to the sample surface in every case. The emission was also collected normal to the sample surface, dispersed in a Triax 550 spectrometer and detected using a Hamamatsu R5509-72 IR-PMT. The erbium emission intensity was measured at 1532 nm. For both lasers the beams were expanded and a uniform illumination of the sample, through a 1 mm diameter hole, was used. The power density of each measurement was measured directly by replacing the sample with a calibrated silicon photodetector. Ten measurements of the erbium emission intensity were recorded for each excitation power density and the average and standard error calculated for each. Each sample measurement was repeated on different days to ensure that the results were reproducible.

All films were flat and uniform with low surface roughness and no evidence of agglomeration of the two molecules.

## Electronic supplementary material

Supplementary Information
