# Supplementary material for: High sensitization efficiency and energy transfer routes for population inversion at low pump intensity in Er organic complexes for IR amplification
Source: Sci Rep. 2018 Feb 19;8:3226. doi: 10.1038/s41598-018-21700-7 (PMC5818663; doi:10.1038/s41598-018-21700-7)
Supplement: Supplementary file 1 — Supplementary Information [file 41598_2018_21700_MOESM1_ESM.docx]

Supplementary information

**Title:** **High sensitization efficiency and energy transfer routes for population inversion at low pump intensity in Er organic complexes for IR amplification**

Jianxu Hu, Svitlana Karamshuk, Jelena Gorbaciova, Huanqing Ye, Haizhou Lu, Yuanpeng Zhang, Youxuan Zheng, Xiao Liang, Ignaico Hernández, Peter B. Wyatt^*^ and William P. Gillin^*^

Figure S1. Chemical structures of compounds used.

H–F-TPIP (free acid) Zn(F-BTZ)_2_

**
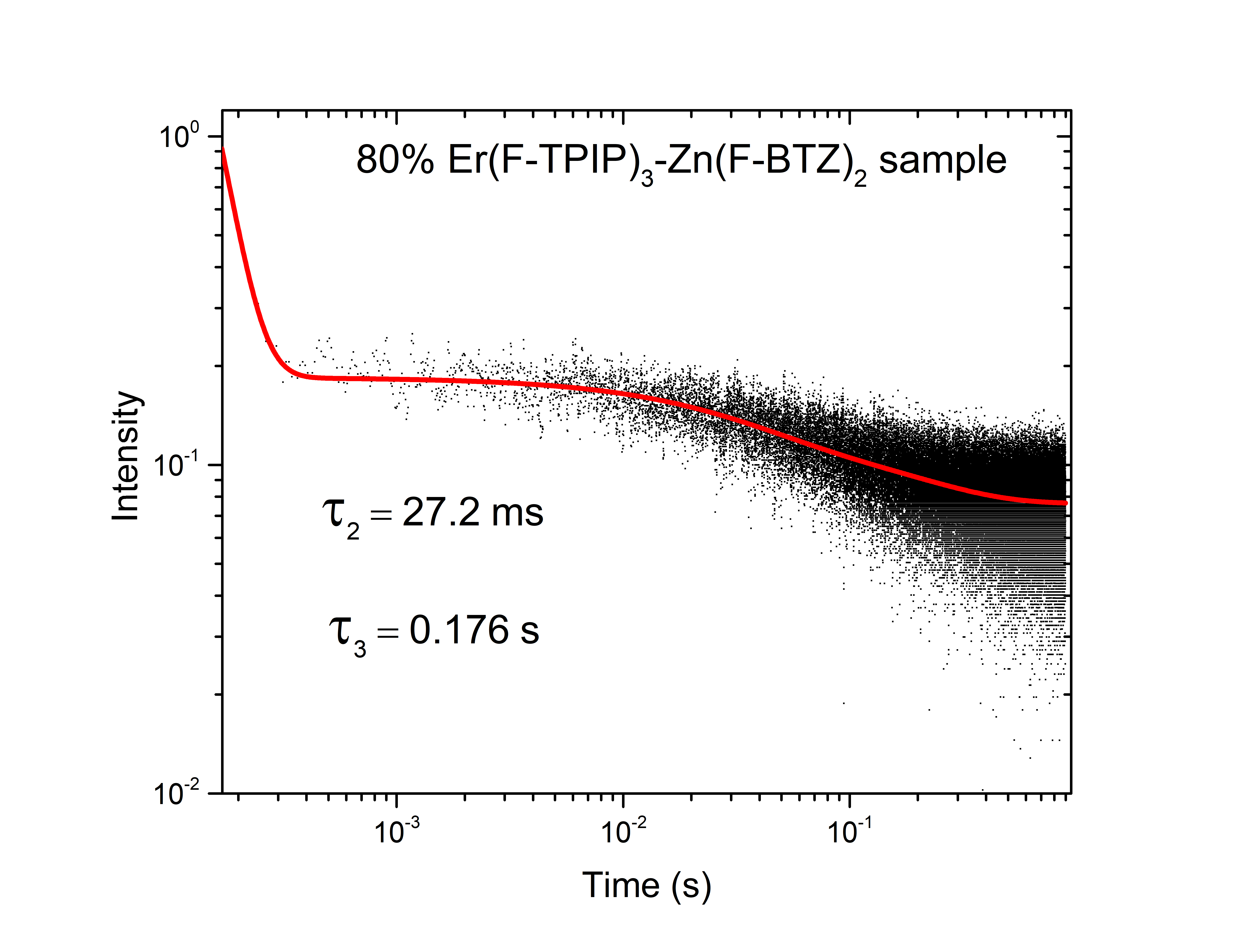
**Figure S2. Typical triplet emission lifetime under 407 nm laser excitation at 80K.

The triplet lifetime measured at 80K shows three distinct lifetime components for all Er(F-TPIP)_3_-Zn(F-BTZ)_2_ films. The fastest lifetime component falls within the range of the instrument response time, which is not shown in Figure S2. The second longest lifetime component is in the range of tens of milliseconds, while the longest lifetime component is in the range of hundreds of milliseconds.


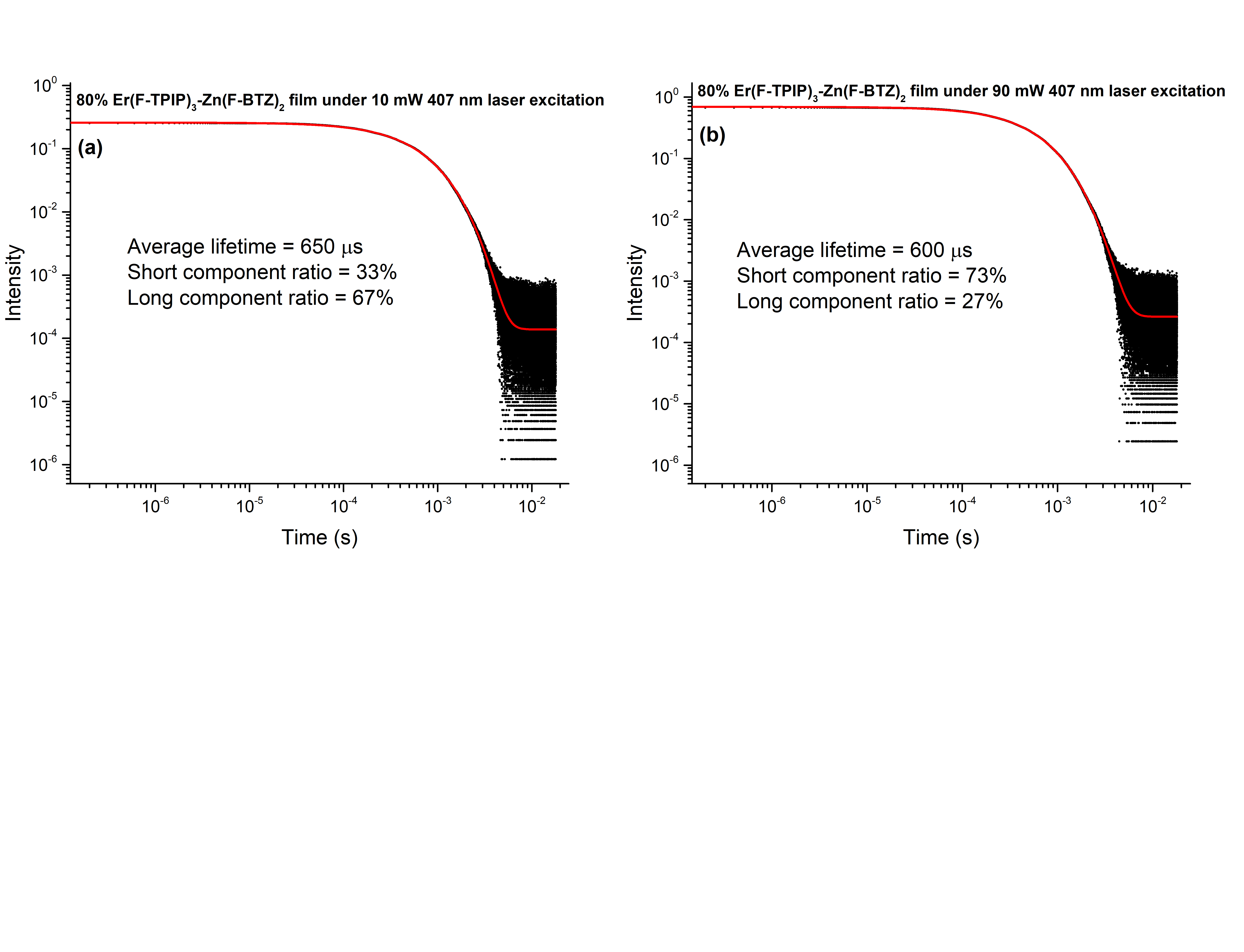
Figure S4. Typical lifetime at 1532 nm under the excitation of 407 nm laser with different powers for Er(F-TPIP)_3_-Zn(F-BTZ)_2_ samples.

The lifetime at 1532 nm of the Er(F-TPIP)_3_-Zn(F-BTZ)_2_ samples shows a bi-exponential characteristic with a long lifetime component (⁓700 μs) and a short lifetime component (⁓450 μs).

Figure S5. AFM image for a 2 µm thick layer of a 43% Er(F-TPIP)_3_ and 57% Zn(F-BTZ)_2_ film.


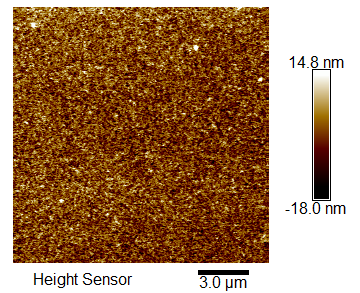


The 2 µm film used for the AFM characterisation is approximately a factor of 5 thicker than those used for the luminescence studies. The film shows a mean surface roughness of 3.6 nm and no evidence of segregation of the two components.

Table S1. Short lifetime component measured by the 407 nm laser for Er(F-TPIP)_3_-Zn(F-BTZ)_2_ samples. The unit for lifetime is microseconds (μs) and the unit for power density is mW/cm^2^.

| Er(F-TPIP)_3_ concentration  Power density | 9% | 23% | 44% | 64% | 80% |
| --- | --- | --- | --- | --- | --- |
| 113.73 | 410±30 | 422±2.2 | 415±48 | 429±24 | 442±11 |
| 335.47 | 428±18 | 436±35 | 436±5.7 | 442±22 | 457±6.8 |
| 567.4 | 429±6.8 | 456±5.1 | 465±5.2 | 457±5.3 | 462±4.2 |
| 790.15 | 431±26 | 468±4.5 | 477±2.2 | 474±3.7 | 487±3.6 |
| 1017.76 | 438±9.5 | 473±19 | 489±4.1 | 491±4.8 | 509±3.8 |

Table S2. Long lifetime component measured by the 407 nm laser for Er(F-TPIP)_3_-Zn(F-BTZ)_2_ samples. The unit for lifetime is microseconds (μs) and the unit for power density is mW/cm^2^.

| Er(F-TPIP)_3_ concentration  Power density | 9% | 23% | 44% | 64% | 80% |
| --- | --- | --- | --- | --- | --- |
| 113.73 | 774±19 | 727±4.2 | 702±4.4 | 694±18 | 676±11 |
| 335.47 | 748±16 | 706±27 | 687±4.7 | 673±8.6 | 662±10 |
| 567.4 | 731±2.9 | 683±2.4 | 678±6.9 | 654±2.9 | 649±6.2 |
| 790.15 | 703±42 | 671±6.1 | 662±7.1 | 647±5.4 | 632±6.6 |
| 1017.76 | 693±3.7 | 658±12 | 652±5.9 | 634±2.3 | 624±5.1 |

**Parameters used in the modelling:**

For the rate equations only three levels are considered: the ground state (^4^I_15/2_) of the Er^3+^, (with a population of *N*_0_), the first excited state (^4^I_13/2_) of the Er^3+^, (with a population of *N*_1_), and the third excited state (^4^F_9/2_) of the Er^3+^, (with a population of *N*_2_)*.* The transition rate, A_20_, (^4^F_9/2_ to ^4^I_15/2_) was taken as 1000 s^-1^ and was obtained from a Judd-Ofelt calculation for Er(F-TPIP)_3_. The transition rate A_21_ (^4^F_9/2_ to ^4^I_13/2_) (τ_21_=0.5 µs) was measured directly from the rise time for the ^4^I_13/2_ to ^4^I_15/2_ transition when the ^4^F_9/2_ level was excited directly using an ~5 ns pulse at 655 nm from an OPO. The initial population of ions in the ground state (*N*_0_) is known from the Er concentration in the films. The value of A_10_ we used in our model was determined from a linear fit to the average lifetime at 1532 nm and hence was a function of the excitation intensity. The pump rate, *R_P_*, is given by the absorption cross-section for the ^4^I_15/2_ to ^4^F_9/2_ (*σ_abs_* = 1.3×10^-21^ cm^2^) multiplied by the photon flux density (*φ*).

Table S3. Number of erbium ions used in the sensitization efficiency modelling for Er(F-TPIP)_3_ doped Zn(FBTZ)_2_ samples.

| Er(F-tpip)_3_ concentration | 9 mol% | 23 mol% | 43 mol% | 64 mol% | 80 mol% |
| --- | --- | --- | --- | --- | --- |
| Er ions in Zn(FBTZ)_2_ samples | 2.58×10^13^ | 7.83×10^13^ | 2.03×10^14^ | 4.69×10^14^ | 1.09×10^15^ |

**Modelling procedures**

Firstly, the population of *N*_1_ under 655 nm laser excitation was obtained using the parameters listed above. Here *R_P_ = σ_abs_φ* as the 655 nm laser only excites erbium ions in the sample. Consequently, the chromophore does not contribute to sensitization in this case and the value of *F_SEN_* is taken as one. Since the signal intensity at 1532 nm under 655 nm laser excitation has been measured, the scaling factor between the population of erbium ions in the first excited state and the corresponding signal intensity can be determined. This scaling factor remains constant for each sample measured due to the identical experimental set-up, regardless of the excitation source.

For the sensitised luminescence under 407 nm excitation we multiply the pump rate by a sensitisation factor *F_SEN_*. This sensitisation factor is then adjusted until the model results fit the experimental data. This then allows us to calculate the excited state population, *N*_1_, and hence the degree of population inversion, as a function of 407 nm excitation.
